# Supplementary material for: Phenol-soluble modulin α and β display divergent roles in mice with staphylococcal septic arthritis
Source: Commun Biol. 2022 Sep 5;5:910. doi: 10.1038/s42003-022-03839-2 (PMC9445034; doi:10.1038/s42003-022-03839-2)
Supplement: Supplementary file 2 — Supplementary Information [file 42003_2022_3839_MOESM2_ESM.pdf]

Supplementary information

Phenol-soluble modulin α and β display divergent roles in mice with staphylococcal septic arthritis

Zhicheng Hu[1,2], Pradeep Kumar Kopparapu[1#], Patrick Ebner[3#], Majd Mohammad[1], Simon Lind[1], Anders Jarneborn[1,4], Claes Dahlgren[1], Michelle Schultz[1], Meghshree Deshmukh[1], Rille Pullerits[1,5], Mulugeta Nega[3], Minh-Thu Nguyen[6], Ying Fei[2], Huamei Forsman[1], Friedrich Götz[3], Tao Jin[1,4*]

* Corresponding author, # Equal contribution

1. Department of Rheumatology and Inflammation Research, Institute of Medicine, Sahlgrenska Academy, University of Gothenburg, Gothenburg, Sweden

2. Center for Clinical Laboratories, the Affiliated Hospital of Guizhou Medical University, Guiyang, China

3. Department of Microbial Genetics, Interfaculty Institute of Microbiology and Infection Medicine Tübingen (IMIT), University of Tübingen, Tübingen, Germany

4. Department of Rheumatology, Sahlgrenska University Hospital, Gothenburg, Sweden

5. Department of Clinical Immunology and Transfusion Medicine, Sahlgrenska University Hospital, Gothenburg, Sweden

6. Institute of Medical Microbiology, University Hospital of Münster, Münster, Germany

E-mail: tao.jin@rheuma.gu.se

Running title: PSMs in *S. aureus* septic arthritis

Supplementary Table 1. Antibodies used in the FACS panel

Antibody	Fluorochrome	Clone	Supplier
Ly6G	PE	1A8	BD
F4/80	PE-Cy7	BM8	Bioscience
Annexin V	FITC		Biolegend
7-aminoactinomycin D (7-AAD)			invitrogen

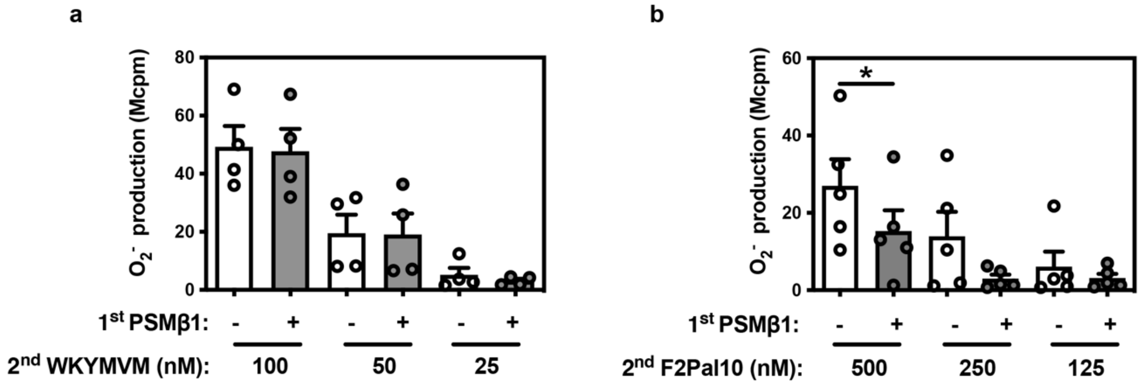

Supplementary Figure 1. PSMβ1 partially inhibits activation of the neutrophil NADPH-oxidase induced by F2Pal10, but not by WKYMVM. NADPH oxidase-induced superoxide anion release (O_2^-, *y*-axis) by neutrophils was measured by isoluminol-amplified chemiluminescence over time (min, *x*-axis). The cells were preincubated at 37 °C for 5 min before being first challenged with PSM β1 and then challenged with a second agonist (WKYMVM or F2Pal10). The peaks of O_2^- release by neutrophils stimulated with PSMβ (500 nM) or buffer control followed by various concentrations of WKYMVM (25-100 nM; n=4) (**a**) or F2Pal10 (125-500 nM; n=5) (**b**) were compared. Statistical comparison was done using paired t test, with data expressed as mean ± stand error of the mean. *$P < 0.05$.

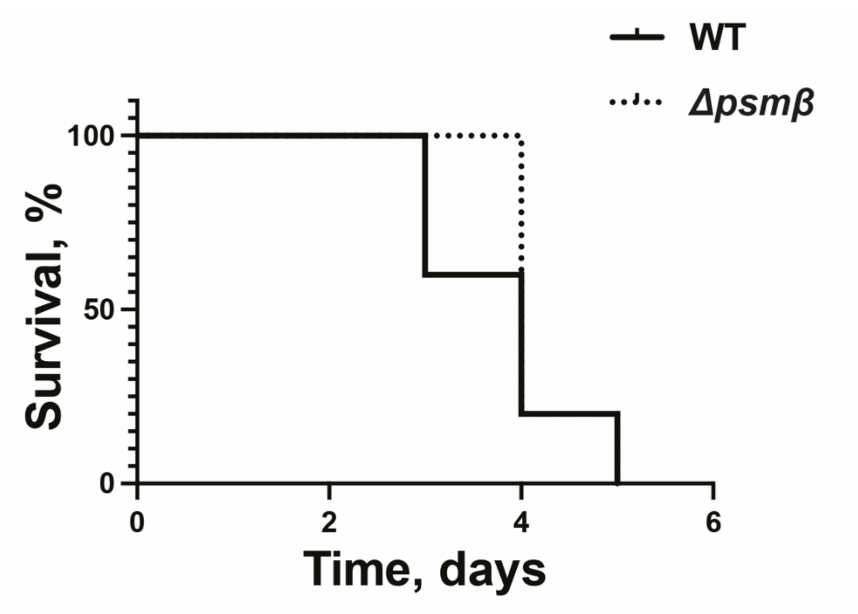

Supplementary Figure 2. PSMβ deficiency had no impact on lethal *S. aureus* sepsis. NMRI mice (n=5/group) were intravenously injected with *S. aureus* Newman (wild type) or Δ*psmβ* mutant (1 x 10^7 colony-forming units [CFU]/mouse) to induce the sepsis. The mortality was followed every 12 hours for 6 days. Statistical analysis was determined by using the log-rank (Mantel-cox) test.

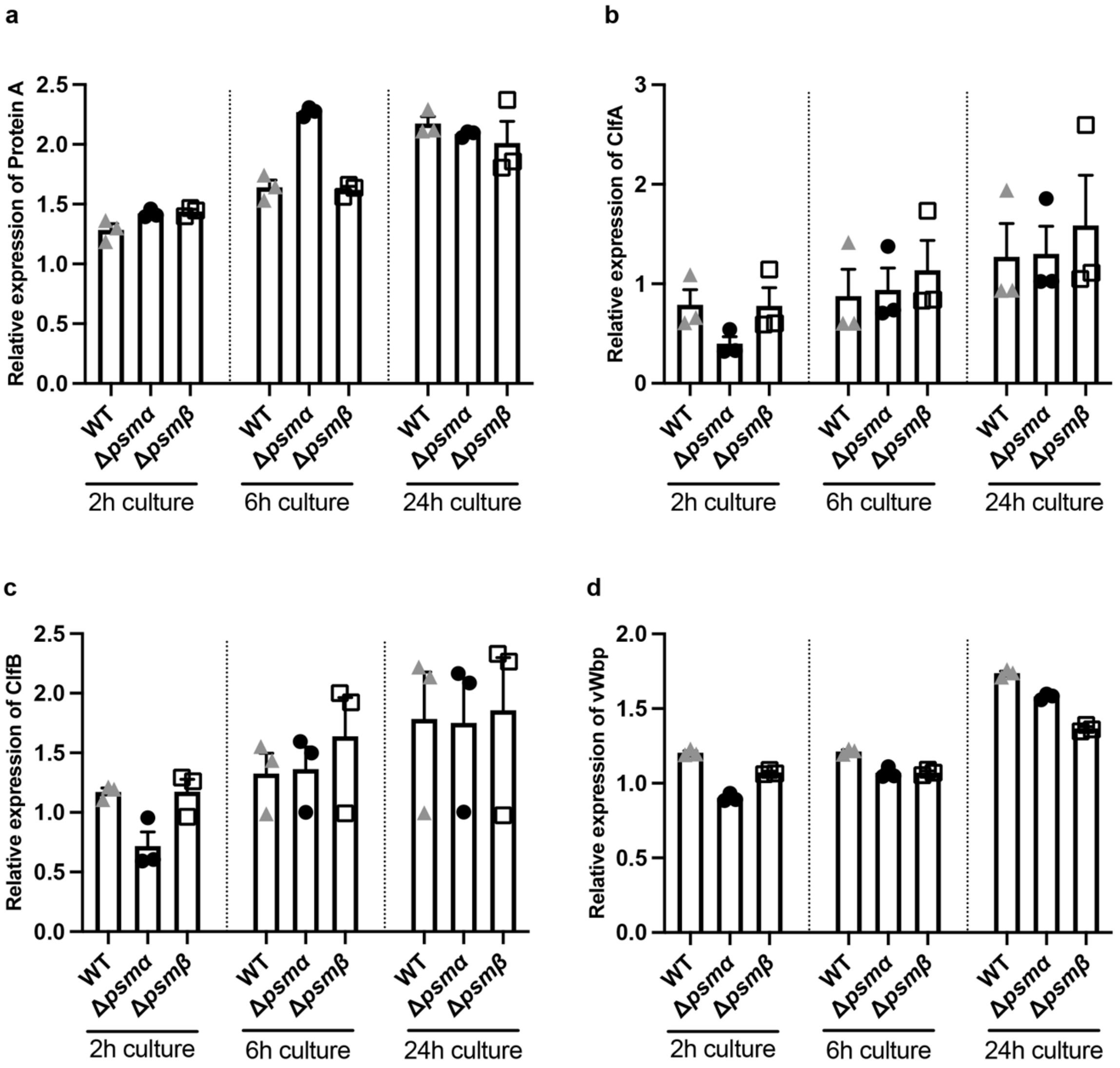

Supplementary Figure 3. Expression of surface proteins in *S. aureus psm* mutants and their parental strain. *S. aureus* Newman (wild type) and Δ*psmα*, Δ*psmβ* mutants were cultured in TSB at 37 ºC for 2, 6 and 24h. The relative gene expression levels of Protein A (Spa) (**a**), Clumping factor A (ClfA) (**b**), Clumping factor B (ClfB) (**c**) and von Willebrand factor-binding protein (vWbp) (**d**) were analyzed with the TaqMan gene expression assays. All samples were run in triplicates and the relative expression was calculated using the ΔΔCt method. Statistical evaluations were performed using the Mann-Whitney *U* test, with data expressed as the mean ± standard error of the mean

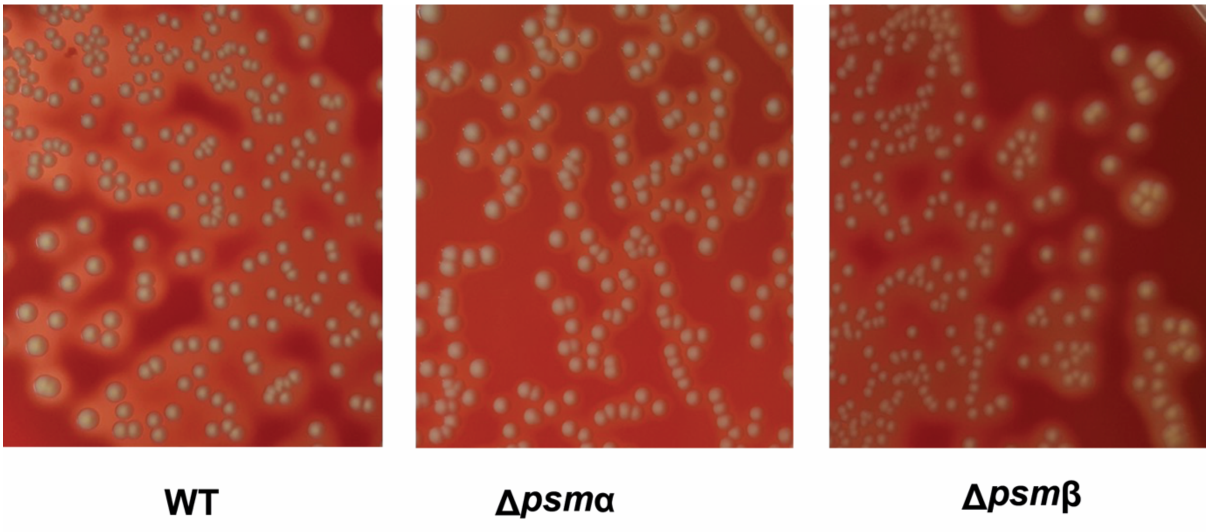

Supplementary Figure 4. Comparative alpha-hemolysis activity in *S. aureus psm* mutants and their parental strain. Hemolysin activity of S. aureus strains was examined on sheep blood agar. *S. aureus* Newman (wild type) and Δ*psmα*, Δ*psmβ* mutants were grown on sheep blood agar at 37 ºC for 18 h, following a cold shock at 4 ºC for 12 h. The hemolysin lysed the erythrocytes of sheep blood cells and created a clear halo.

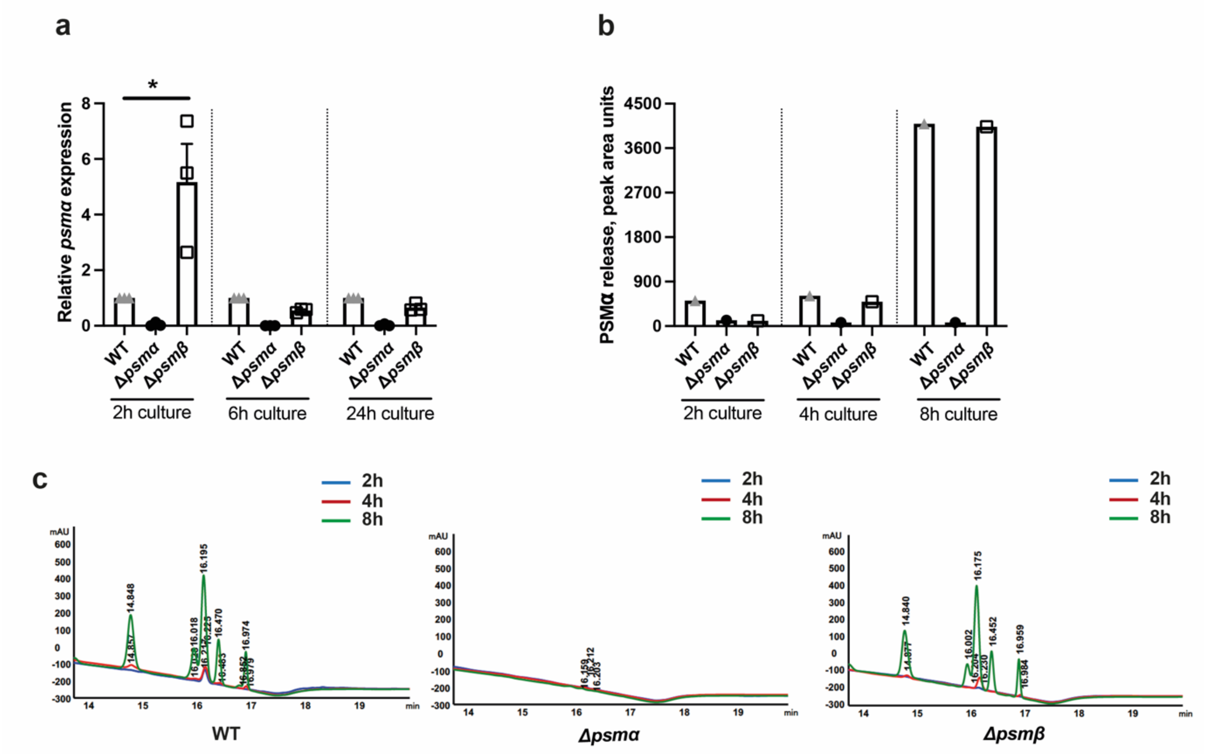

Supplementary Figure 5. PSMα release profile in *S. aureus* PSM mutants and their parental strain. *S. aureus* Newman (wild type) and Δ*psmα*, Δ*psmβ* mutants were cultured in TSB at 37 ºC for 24 hours. The relative gene expression of *psmα* was analyzed by RT-PCR and the PSMα levels in culture medium were analyzed by HPLC. (**a**) The relative gene expression levels of PSMα in those strains at 2, 6, and 24 hours of culture. (**b**) PSMα levels in culture medium by Newman (wild type) and Δ*psmα*, Δ*psmβ* mutants at 2, 4, and 8 hours of culture. (**c**) HPLC profiles of PSMα in *S. aureus* Newman WT and its corresponding *psmα* and *psmβ* deletion mutants at 2, 4, and 8 hours of culture. Statistical evaluations were performed using the unpaired t test, with data expressed as the mean ± standard error of the mean (a). *$P < 0.05$.
