# Supplementary material for: Phenol-soluble modulin α and β display divergent roles in mice with staphylococcal septic arthritis
Source: Commun Biol. 2022 Sep 5;5:910. doi: 10.1038/s42003-022-03839-2 (PMC9445034; doi:10.1038/s42003-022-03839-2)
Supplement: Supplementary file 3 — Description of Additional Supplementary Files [file 42003_2022_3839_MOESM3_ESM.pdf]

Description of Additional Supplementary Files

File name: Supplementary Data 1
Description: Sheet (1) Raw data for figure 1a: O2− production level. Sheet (2) Raw data for figure 1b: O2− production level(peak value). Sheet (3) Raw data for figure 1c: O2− production level. Sheet (4) Raw data for figure 1d: Percentage of remaining activity.

File name: Supplementary Data 2
Description: Sheet (1) Raw data for figure 2a: O2− production level. Sheet (2) Raw data for figure 2b: O2− production level(peak value). Sheet (3) Raw data for figure 2c: O2− production level(peak value).

File name: Supplementary Data 3
Description: Sheet (1) Raw data for figure 3a: O2− production level. Sheet (2) Raw data for figure 3b: O2− production level (peak value).

File name: Supplementary Data 4
Description: Sheet (1) Raw data for figure 4a: Clinical arthritis index. Sheet (2) Raw data for figure 4b: Clinical arthritis frequency. Sheet (3) Raw data for figure 4c: Clinical polyarthritis frequency. Sheet (4) Raw data for figure 4d: Weight development.

File name: Supplementary Data 5
Description: Sheet (1) Raw data for figure 5a: Bone erosion score. Sheet (2) Raw data for figure 5b: Bone erosion frequency.

File name: Supplementary Data 6
Description: Sheet (1) Raw data for figure 6a: Kidney abscess scores. Sheet (2) Raw data for figure 6b: Bacterial counts in kidneys. Sheet (3) Raw data for figure 6c: Persistence of bacterial frequency in joints.

File name: Supplementary Data 7
Description: Sheet (1) Raw data for figure 7a: ELISA data for IL-6. Sheet (2) Raw data for figure 7b: ELISA data for KC.

File name: Supplementary Data 8
Description: Sheet (1) Raw data for figure 8b-e: FACS data.
