# Supplementary material for: Phenol-soluble modulin α and β display divergent roles in mice with staphylococcal septic arthritis
Source: Commun Biol. 2022 Sep 5;5:910. doi: 10.1038/s42003-022-03839-2 (PMC9445034; doi:10.1038/s42003-022-03839-2)
Supplement: Supplementary file 12 — Reporting Summary [file 42003_2022_3839_MOESM12_ESM.pdf]

nature portfolio

Corresponding author(s): Tao Jin
tao.jin@rheuma.gu.se

Last updated by author(s): Aug 11, 2022

Reporting Summary

Nature Portfolio wishes to improve the reproducibility of the work that we publish. This form provides structure for consistency and transparency in reporting. For further information on Nature Portfolio policies, see our Editorial Policies and the Editorial Policy Checklist.

Statistics

For all statistical analyses, confirm that the following items are present in the figure legend, table legend, main text, or Methods section.

n/a	Confirmed	
☐	☒	The exact sample size (*n*) for each experimental group/condition, given as a discrete number and unit of measurement
☐	☒	A statement on whether measurements were taken from distinct samples or whether the same sample was measured repeatedly
☐	☒	The statistical test(s) used AND whether they are one- or two-sided<br>*Only common tests should be described solely by name; describe more complex techniques in the Methods section.*
☒	☐	A description of all covariates tested
☒	☐	A description of any assumptions or corrections, such as tests of normality and adjustment for multiple comparisons
☐	☒	A full description of the statistical parameters including central tendency (e.g. means) or other basic estimates (e.g. regression coefficient) AND variation (e.g. standard deviation) or associated estimates of uncertainty (e.g. confidence intervals)
☒	☐	For null hypothesis testing, the test statistic (e.g. *F*, *t*, *r*) with confidence intervals, effect sizes, degrees of freedom and *P* value noted<br>*Give P values as exact values whenever suitable.*
☒	☐	For Bayesian analysis, information on the choice of priors and Markov chain Monte Carlo settings
☒	☐	For hierarchical and complex designs, identification of the appropriate level for tests and full reporting of outcomes
☒	☐	Estimates of effect sizes (e.g. Cohen's *d*, Pearson's *r*), indicating how they were calculated

Our web collection on statistics for biologists contains articles on many of the points above.

Software and code

Policy information about availability of computer code

Data collection	N/A
Data analysis	N/A

For manuscripts utilizing custom algorithms or software that are central to the research but not yet described in published literature, software must be made available to editors and reviewers. We strongly encourage code deposition in a community repository (e.g. GitHub). See the Nature Portfolio guidelines for submitting code & software for further information.

Data

Policy information about availability of data

All manuscripts must include a data availability statement. This statement should provide the following information, where applicable:

- Accession codes, unique identifiers, or web links for publicly available datasets
- A description of any restrictions on data availability
- For clinical datasets or third party data, please ensure that the statement adheres to our policy

The authors declare that the main data supporting the findings of this study are available within the article and its Supplementary Information files. Extra data are available from the corresponding author upon request.

Human research participants

Policy information about studies involving human research participants and Sex and Gender in Research.

Reporting on sex and gender	N/A
Population characteristics	N/A
Recruitment	N/A
Ethics oversight	N/A

Note that full information on the approval of the study protocol must also be provided in the manuscript.

Field-specific reporting

Please select the one below that is the best fit for your research. If you are not sure, read the appropriate sections before making your selection.

☒ Life sciences ☐ Behavioural & social sciences ☐ Ecological, evolutionary & environmental sciences

For a reference copy of the document with all sections, see nature.com/documents/nr-reporting-summary-flat.pdf

Life sciences study design

All studies must disclose on these points even when the disclosure is negative.

Sample size	The sample sizes were chosen or calculated based on the results from previous experiments.
Data exclusions	No data was excluded.
Replication	The experiments were repeated at least two times. All attempts at replication were successful.
Randomization	Yes, animals in all experiments were randomized.
Blinding	All investigators were blinded to the treatment groups.

Reporting for specific materials, systems and methods

We require information from authors about some types of materials, experimental systems and methods used in many studies. Here, indicate whether each material, system or method listed is relevant to your study. If you are not sure if a list item applies to your research, read the appropriate section before selecting a response.

Materials & experimental systems

n/a	Involved in the study	
☐	☒	Antibodies
☒	☐	Eukaryotic cell lines
☒	☐	Palaeontology and archaeology
☐	☒	Animals and other organisms
☒	☐	Clinical data
☒	☐	Dual use research of concern

Methods

n/a	Involved in the study	
☒	☐	ChIP-seq
☐	☒	Flow cytometry
☒	☐	MRI-based neuroimaging

Antibodies

Antibodies used	The following antibodies were used: PE Rat Anti-Mouse Ly-6G (551461; Clone: 1A8; BD); Anti-Mouse F4/80 (25-4801-82; Clone: BM8; Bioscience Inc.); FITC-conjugated Annexin V (640906; Biolegend); 7-aminoactinomycin D (7-AAD, A1310; invitrogen)
Validation	The different antibodies have each been validated previously. Please see below:<br>PE Rat Anti-Mouse Ly-6G and Anti-Mouse F4/80: Fine et al, 2019 (PMID: 31138591)<br>Annexin V and 7-aminoactinomycin D: Molhoek et al, 2009 (PMID: 18841360)

Animals and other research organisms

Policy information about studies involving animals; ARRIVE guidelines recommended for reporting animal research, and Sex and Gender in Research

Laboratory animals	Female NMRI mice, aged 6-12 weeks, were purchased from Envigo (Venray, Netherlands)
Wild animals	N/A
Reporting on sex	N/A
Field-collected samples	N/A
Ethics oversight	Mouse studies were reviewed and approved by the Ethics Committee of Animal Research of Gothenburg. Mouse experiments were conducted in accordance with recommendations listed in the Swedish Board of Agriculture's regulations and recommendations on animal experiments.

Note that full information on the approval of the study protocol must also be provided in the manuscript.

Flow Cytometry

Plots

Confirm that:

☒ The axis labels state the marker and fluorochrome used (e.g. CD4-FITC).

☒ The axis scales are clearly visible. Include numbers along axes only for bottom left plot of group (a 'group' is an analysis of identical markers).

☒ All plots are contour plots with outliers or pseudocolor plots.

☒ A numerical value for number of cells or percentage (with statistics) is provided.

Methodology

Sample preparation	Whole blood was collected into EDTA coated tube. For 1 mL whole blood, 9 mL of eBioscience™ 1X RBC Lysis Buffer (Invitrogen, Waltham, MA, US) was added for red blood cells (RBCs) lysis, after 10 minutes, cells were centrifuged and resuspended in flow-assisted cell sorting (FACS) buffer (3% Hi FCS, 1 mM EDTA), 2 million cells were blocked with 2 μL Mouse BD FcBlock™ (BD Biosciences) for 5 minutes on ice, cells were resuspended in antibody cocktail for 20 minutes after centrifugation, then, washed twice with cold PBS and stained with FITC Annexin V kit (Biolegend, San Diego, CA, US) and 7-aminoactinomycin D (7-AAD; Invitrogen, Waltham, MA, US) according to manufacturer's instructions.
Instrument	Cells were acquired on a BD FACSLyric flow cytometer (BD Biosciences).
Software	Data was analyzed using FlowJo version 10.8 software (Tree Star, Ashland, USA).
Cell population abundance	N/A
Gating strategy	The gating strategy was provided in Figure 8.

☐ Tick this box to confirm that a figure exemplifying the gating strategy is provided in the Supplementary Information.
